# Next generation phenotyping using narrative reports in a rare disease clinical data warehouse

**DOI:** 10.1186/s13023-018-0830-6

**Published:** 2018-05-31

**Authors:** Nicolas Garcelon, Antoine Neuraz, Rémi Salomon, Nadia Bahi-Buisson, Jeanne Amiel, Capucine Picard, Nizar Mahlaoui, Vincent Benoit, Anita Burgun, Bastien Rance

**Affiliations:** 1 Institut Imagine, Paris Descartes Paris Descartes-Sorbonne Paris Cité University, Paris, France; 2 Institut National de la Santé et de la Recherche Médicale (INSERM), Centre de Recherche des Cordeliers, UMR 1138 Equipe 22, Paris Descartes, Sorbonne Paris Cité University, Paris, France; 3 Department of Medical Informatics, Necker-Enfants Malades Hospital, Assistance Publique des Hôpitaux de Paris (AP-HP), Paris, France; 4 Pediatric Nephrology, Necker Enfants Malades Hospital AP-HP, Université Paris Descartes, Paris, France; 5 Pediatric Neurology, Necker Enfants Malades Hospital AP-HP, Université Paris Descartes, Paris, France; 6 Laboratory of embryology and genetics of congenital malformations, INSERM UMR 1163, Institut Imagine, Paris, France; 7 Department of Genetic, Necker Enfants Malades Hospital AP-HP, Université Paris Descartes, Paris, France; 8 Laboratory of Lymphocyte Activation and Susceptibility to EBV infection, INSERM UMR 1163, Paris Descartes Sorbonne Paris Cité University, Imagine Institute, Paris, France; 9 Study center for primary immunodeficiencies (CEDI) Necker Enfants Malades Hospital AP-HP, Université Paris Descartes, Paris, France; 10 French National Reference Center for Primary Immuno Deficiencies (CEREDIH), Necker Enfants Malades Hospital AP-HP, Université Paris Descartes, Paris, France; 11 Pediatric Immuno-Haematology and Rheumatology Necker Enfants Malades Hospital AP-HP, Université Paris Descartes, Paris, France; 12 Hôpital Européen Georges Pompidou, AP-HP, Université Paris Descartes, Sorbonne Paris Cité, Paris, France; 13 Imagine - Institute of Genetic Diseases, 24 boulevard du Montparnasse, 75015 Paris, France

**Keywords:** Data warehouse, Next generation phenotyping, Data mining, Rare diseases, Natural language processing

## Abstract

**Background:**

Secondary use of data collected in Electronic Health Records opens perspectives for increasing our knowledge of rare diseases. The clinical data warehouse (named Dr. Warehouse) at the Necker-Enfants Malades Children’s Hospital contains data collected during normal care for thousands of patients. Dr. Warehouse is oriented toward the exploration of clinical narratives. In this study, we present our method to find phenotypes associated with diseases of interest.

**Methods:**

We leveraged the frequency and TF-IDF to explore the association between clinical phenotypes and rare diseases. We applied our method in six use cases: phenotypes associated with the Rett, Lowe, Silver Russell, Bardet-Biedl syndromes, DOCK8 deficiency and Activated PI3-kinase Delta Syndrome (APDS). We asked domain experts to evaluate the relevance of the top-50 (for frequency and TF-IDF) phenotypes identified by Dr. Warehouse and computed the average precision and mean average precision.

**Results:**

Experts concluded that between 16 and 39 phenotypes could be considered as relevant in the top-50 phenotypes ranked by descending frequency discovered by Dr. Warehouse (resp. between 11 and 41 for TF-IDF). Average precision ranges from 0.55 to 0.91 for frequency and 0.52 to 0.95 for TF-IDF. Mean average precision was 0.79. Our study suggests that phenotypes identified in clinical narratives stored in Electronic Health Record can provide rare disease specialists with candidate phenotypes that can be used in addition to the literature.

**Conclusions:**

Clinical Data Warehouses can be used to perform Next Generation Phenotyping, especially in the context of rare diseases. We have developed a method to detect phenotypes associated with a group of patients using medical concepts extracted from free-text clinical narratives.

**Electronic supplementary material:**

The online version of this article (10.1186/s13023-018-0830-6) contains supplementary material, which is available to authorized users.

## Background

The global trend toward digital health in the US and in Europe has led to an unprecedented adoption of Electronic Health Records (EHRs). By the end of 2014, 83% of US physicians [1] and 75% of hospitals [2] used some form of EHRs. The increasing number of EHRs opens strong perspectives for the secondary use of data collected during the care process. Many hospitals are now equipped with Clinical Data Warehouses (CDW) integrating all the data produced during the care of the patients for research purposes [3–5]. CDWs gather a large variety of information, ranging from structured data (e.g. diagnosis codes, laboratory test results…) to free-text clinical narratives and images. Structured data include coded data using terminologies like the International Classification of Diseases, and questionnaires that provide precise, standardized but somehow limited information. Conversely free-text reports are produced without constraints and may be used to express nuanced, unexpected, and unexplained signs or symptoms regarding the patient case. Clinical narratives collect information from all aspects of the patient care that might not be collected anywhere else in clinical information system including history of the disease, family history, fine-grained description of all the symptoms, hypothesis of diagnosis or treatment, information from treatments received outside of the hospital, and so forth. Previous studies in different contexts showed the importance of free-text in EHRs. For example Raghavan et al. identified that unstructured data were essential to solve trial criteria from two studies*.* [6]. The value of text data is even more important to detect phenotypes in specialized hospitals treating patients with rare diseases and for outpatients, for whom clinical information is barely coded [7].

Rare diseases represent a large group of heterogeneous conditions and some cases remain undiagnosed for a long time. A precise phenotypic description of such diseases can be problematic given the small number of cases and the heterogeneity of the phenotypes. Leveraging large CDWs, could be helpful to enrich this description. While structured (standardized) questionnaires exist for several rare diseases (e.g., in France [8, 9]), part of the clinical description is still present only in free text in EHRs. We hypothesized that mining large collections of clinical texts in hospitals specialized in rare diseases could offer interesting perspectives to enrich the descriptions provided by dedicated knowledge bases. We investigated this hypothesis at the *Necker Enfants Malades Hospital* (Necker Children Hospital), a children’s hospital in Paris that is associated with the *Imagine* research institute, specialized in genetic diseases, and hosts 15 national reference centers for rare diseases. We illustrate our approach on six rare diseases: DOCK8 deficiency, the Activated PI3-kinase Delta Syndrome (APDS), Rett, Lowe, Silver Russell and Bardet Biedl syndromes. The combined immunodeficiency due to DOCK8 deficiency (prevalence less than 1/1,000,000) is a form of autosomal recessive combined immunodeficiency (T, B and NK cells), characterized by recurrent lung infections, cutaneous viral infections, allergy, severe skin inflammation and susceptibility to cancer with a high level of IgE [10]. DOCK8 deficiency is caused by homozygous or compound heterozygous mutations in *DOCK8* gene [11].

The activated phosphoinositide 3-kinase-δ (PI3Kδ) syndrome (APDS) (estimated prevalence < 1 /1,000,000) is characterized by immunodeficiency and recurrent respiratory tract infections, lymphoproliferation and hypogammaglobulinemia. APDS is caused by activating heterozygous mutations in *PIK3CD* (APDS1) or in *PIK3R1* (APDS2) [11, 12].

Rett syndrome (estimated prevalence 1/15,000) is characterized by a rapid regression in language and motor skills (i.e. repetitive, stereotypic hand movements) after six to eighteen months of normal psychomotor development [13].

The Lowe syndrome or Oculocerebrorenal syndrome (estimated prevalence 1 to 9 /1,000,000) is a multisystem disorder characterized by congenital cataract, intellectual disabilities, glaucoma, postnatal growth retardation and renal tubular dysfunction [14].

The Silver-Russell syndrome (prevalence 1–9 /1,000,000) is characterized by growth retardation with antenatal onset, characteristic facies and limb asymmetry [15].

The Bardet-Biedl syndrome (prevalence estimated at 1 to 9 /1,000,000) is a ciliopathy characterized by a combination of clinical signs including obesity, pigmentary retinopathy, post-axial polydactyly, polycystic kidneys [16].

From now on, we will refer to as phenotype any sign or symptom, disease, defects, and so forth, affecting a patient.

In this study, we present the methods that we developed to extract phenotypes associated with rare diseases from clinical texts in Dr. Warehouse® (DrWH), the clinical data warehouse of the Necker Children’s hospital. Then, we evaluate the scalability of our approach in the context of high throughput phenotyping.

### Material

All data were collected from the *Necker Enfants Malades Hospital* (Necker Children Hospital), a pediatric University hospital belonging to the Assistance Publique Hôpitaux de Paris group (400 pediatric beds, 200 adult beds). The Necker hospital is a national reference center for rare and undiagnosed diseases. The hospital hosts the *Imagine Institute*, a research institute focused on genetic diseases. *Imagine institute* has been developing since 2015 a document-based open-source clinical data warehouse oriented toward free-text: Dr. Warehouse® (DrWH). DrWH includes a full text search engine, and contains, as of August 2017 more than 3.9 million clinical free-text documents for more than 446,000 patients.

In Table 1, we describe the demographic characteristics of the patients included in DrWH. We used all the clinical narratives, ranging from hospitalization to outpatient visits reports, available in DrWH to perform this study. The heterogeneity of the records is illustrated in Table 2 with the distribution of these records by hospital departments and type of reports.

**Table 1:** Description of the population of the data warehouse at Necker hospital

	DrWH
Nb patients	446,481
Sex ratio (M)	47%
Median Nb reports excluding biological reports per patient	2 [1–6]
Median follow up (years) per patient	0.06 [0–2]

In brackets lower and upper quartile

**Table 2:** Number of documents per Hospital department and per type of records

Hospital departments	# Documents	Types of records	# Documents
Gyneco-Obstetrics	433,698	Laboratory	1,563,450
Pediatric Cardiology	253,474	Consultation	834,619
Adult Clinical Hematology	227,520	Imaging	379,538
Metabolism-Pediatric Neurology	207,804	Discharge letter	293,342
Nephrology Transplantations Adult	187,388	Diagnostic Related Group	255,312
Pediatric Nephrology	175,041	Hospitalization	226,723
Pediatric Immuno-Hematology	152,226	surgery	111,598
Pediatric Radiology	151,811	Day hospital	88,244
Adult Radiology	150,612	Emergency	41,515
Pediatric Cardiac Surgery	136,272	Exams	31,042
Pediatric Visceral Surgery	121,758	Prescription	24,859
Pediatric Orthopedic Surgery	120,287	Medical certificate	24,222
Adult Nephrology	116,602	Pathology report	24,215
Anesthesia intensive care unit Adult And Pediatric	114,773	Foetopathology	8858
Pediatric Gastroenterology	113,857	Multidisciplinary consultation meeting	6605
Emergency	108,367	Other	5786
General Pediatrics	97,831	Staff meeting reports	3669
Physiology	88,981	Total	3,923,597
Pediatric ear nose and throat	82,717		
Pediatric Intensive Care Unit	77,599		
Other	804,979		
Total	3,923,597		

A demonstration version of DrWH is publicly available at the URL: https://imagine-plateforme-bdd.fr/dwh_pubmed/. Note that for privacy reasons, this demo version has been populated with data from PubMed abstracts and not with patient data.

To represent the phenotypes, we used the terminologies from the Unified Medical Language System® (UMLS [17]). The UMLS is considered the lingua franca of medical vocabularies. The UMLS has a large coverage of biomedical vocabularies mostly in English. The UMLS is assembled by integrating 153 medical vocabularies, including generalist terminologies (e.g. MeSH or SNOMED CT), or specialized ones (e.g. the Human Phenotype Ontology - HPO, OMIM, the Gene Ontology). The UMLS Metathesaurus® contains about 3.2 million concepts identified by their unique identifier (the Concept Unique Identifier: CUI). A concept is a cluster of synonymous terms coming from various source vocabularies (13+ million of synonymous terms). In the UMLS the creation of concepts is semi-automatic. For example, the Rett Syndrome CUI is C0035372, this concept is made of terms provided by 145 terms from 50 terminologies. Hierarchical relations or other types of relations are extracted from the source terminologies and included in the UMLS. The UMLS Semantic Network is a much smaller network of 133 semantic types (e.g. Disease or Syndrome, Anatomical Abnormality…). Each Metathesaurus concept is assigned at least one semantic type. The UMLS integrates mostly terms in English, but other language such as French have a non-negligible coverage (397,203 terms).

Our source for reference data was Orphanet, an online resource gathering and integrating knowledge on rare diseases. Orphanet was established in France in 1997 and became a European initiative now involving a consortium of 40 countries in Europe and the rest of the world. Orphanet data are organized using ontologies and structured data [18]. Orphadata is a partial extraction of the data stored in Orphanet freely accessible and organized as XML files [19]. Orphanet proposes a vocabulary for rare diseases. Experts and terminologists have identified synonymous terms associated with disease. Orphanet also provides mappings between Orphanet concepts and a variety of other terminologies (e.g. HPO) to enable interoperability.

Orphanet is dedicated to a specific domain, much narrower than the UMLS but highly specialized and manually curated. In addition, the Orphanet vocabulary has been translated into other languages (including French). The HPO is integrated with the UMLS (as terms of UMLS concepts), and is mapped to Orphanet concepts. Therefore, HPO can serve as a pivot between the two vocabularies.

All the terminologies described above are mainly constituted of English terms, see the related work section of the discussion for further comments on non-English text processing.

## Methods

In this study, we aim at using automated methods to extract phenotypes from the narrative reports. For this purpose, we mined the large body of text documents available in the CDW. This section describes the free-text document processing to automatically extract phenotypes from the narrative reports, and details the exploration of phenotypes associated with six use cases.

### Processing text-documents.

In a nutshell, we leveraged the UMLS to extract phenotypical terms from patients’ text reports. We selected the 397,203 terms (including synonyms) available in French in the UMLS Metathesaurus (version 2017AA) and filtered out terms having less than three characters, or more than 80 characters. To limit the concepts extraction to a phenotypic description, we considered only the concepts assigned to one of the following semantic types: ‘Sign or Symptom’, ‘Disease or Syndrome’, ‘Finding’, ‘Pathologic Function’, ‘Congenital Abnormality’, ‘Physiologic Function’, ‘Anatomical Abnormality’, ‘Neoplastic Process’, ‘Acquired Abnormality’ and ‘Mental or Behavioral Dysfunction’. Finally, we obtained 91,533 terms. In the remainder of the manuscript, we will refer to these terms as phenotypical concepts or UMLS concepts.

**Fig. 1:**
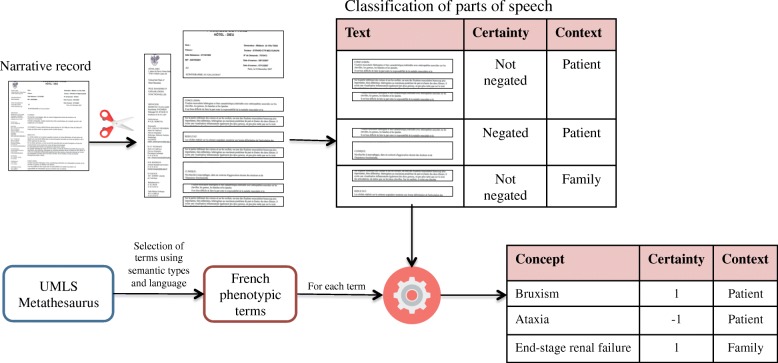
Overview of the method applied to extract phenotypes from the narrative reports

We extracted the phenotypes from every text reports through simple terms matching, case insensitive, and insensitive to non-alphanumerical characters (e.g. spaces, parenthesis, dash etc.). In the context of rare and undiagnosed diseases, clinical narratives are likely to contain many sentences expressing the absence of phenotypes (e.g. “Clinical examination does not support a finding of lupus”, “absence of diabetes”) or describing the family history of the patient (e.g. “the mother has asthma”). Therefore, detecting negation and family history context was essential to exclude these phenotypes from the high throughput phenotyping. We used trigger terms to determine if a phenotype was associated to negated meaning (e.g. “none”, “absence” etc.) or family history context (e.g. “cousin”, “brother”, “sister” etc.). To compute this extraction, we developed an algorithm similar to Context [20, 21], and adapted to French [22] (see Fig. 1).

In this study, we considered exclusively the *not negated* phenotypes associated with the patients (i.e. not associated with their family).

### Use cases: Exploring phenotypes of rare disease patients

**Fig. 2:**
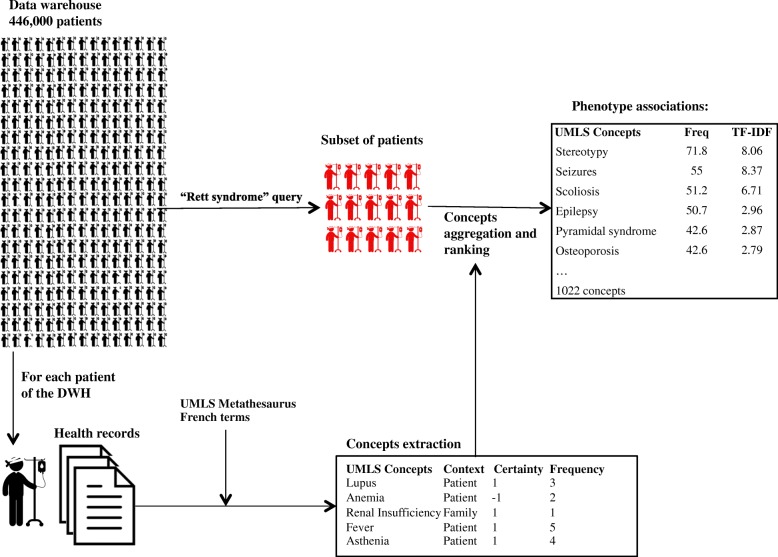
Overview of the method applied to perform next generation phenotyping

We created six groups of patients associated with a specific disease. We queried DrWH at Necker hospital using *Rett Syndrome (and not atypical Rett syndrome), Lowe, Silver Russell, Bardet Biedl, DOCK8 deficiency and APDS* as search criteria. We obtained six sets of patients and their associated corpora of clinical documents (*RETT set, LOWE set, SILVER RUSSELL set, BARDET BIEDL set, DOCK8 deficiency set, and APDS set)*. For each patient set, we extracted all the phenotypes as detailed in the previous section (see Fig. 2).

To rank the extracted phenotypical concepts in terms of relevance, we used two metrics (the frequency and the “term frequency–inverse document frequency” - TF-IDF) classically used in the context of information retrieval. For example, our method identified 1022 distinct phenotypical concepts in the “RETT syndrome” set.

Computing Frequency and TF-IDF:

- The *frequency*: the frequency of the phenotypical concept of interest in the results. For example, the frequency of the term *stereotypy* in the “Rett syndrome” set is 150 (number of patients having at least one mention of *stereotypy* in at least one document) / 209 (number of patients in the set) = 71.8%.
- The *TF-IDF* (term frequency – inverse document frequency) is intended to reflect how important a phenotypical concept is to a patient set in the entire data warehouse. The intuition is that the more frequent is a phenotype in the population, the less specific it is for a given patient set. Conversely finding several occurrences of a rare phenotypical concept in a single patient set highlights the potential interest of this term for this data set. For example, the TF-IDF of the concept *stereotypy* in the “Rett syndrome” result set is 0.081 and is computed as follows:

$$ TF- IDF\ (c)=\frac{N_c}{N_{tot}}\times \log \left(\frac{P_{tot}}{P_c}\right) $$

N_c_: Number of times this phenotypical concept c is used in the set.

N_tot_: Number of not distinct phenotypical concepts in the set.

P_tot_: Total number of patients in the DWH with phenotypical concepts extracted.

P_c_: Number of patients with phenotypical concept c in the set

$$ TF- IDF\ \left(\mathrm{Stereotypy}\right)=\frac{649}{\mathrm{18,538}}\times \log \left(\frac{\mathrm{446,481}}{\mathrm{2,233}}\right)=0.081 $$

### Evaluation

#### Manual evaluation

We considered six use cases. For each of them, a domain expert was asked to browse the highest ranked phenotypes (top-50 phenotypical concepts) found by DrWH and evaluate their relevance with regard to the disease of interest. We presented each expert with two lists of top-50 phenotypes: (i) the top-50 phenotypes ranked by descending frequency and (ii) the top-50 phenotypes ranked by descending TF-IDF. The experts classified the phenotypes as relevant or not relevant to the disease.

We stored the number of relevant phenotypes, and their associated ranks. Based on the experts’ feedbacks, we computed the Average Precision for each query, and the overall Mean Average Precision. The average precision expresses the correctness of the top ranked results for a query. The Mean Average Precision evaluates the average precision across a series of queries [23].

#### Comparison to Orphadata

For each disease set we compared the phenotypical concepts obtained by our method with those in Orphadata with the following steps. We leveraged HPO to map Orphadata and the UMLS (Orphanet is mapped to HPO, and HPO is integrated in the UMLS). We calculated the number of equivalent phenotypical concepts and the number of phenotypical concepts present in only one of the data sources (i.e. DrWH or Orphadata). The phenotypical concepts were considered equivalent (i) in case of exact mapping (same identifier) or (ii) when a broader phenotype was found (in Orphadata: Arrhythmia, in our extraction: Cardiac flutter).

**Fig. 3:**
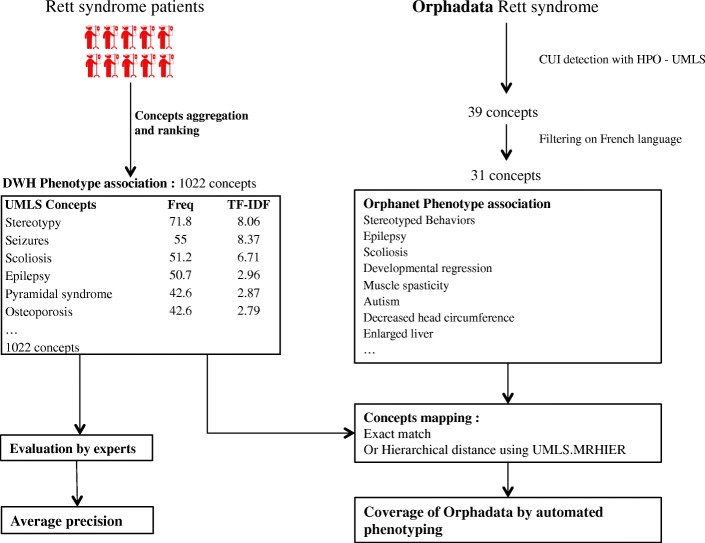
Evaluation procedure for the RETT set

The steps are illustrated with the example of Rett syndrome in Fig. 3.

## Results

### Document processing in DrWH

We extracted a total of 18.7 million phenotypical terms from 3.9 million medical records, representing 446,481 distinct patients. Among these terms, 4% were related to family history. Among the 96% of the remaining terms, 72% were classified into as not negated expression (12.99 million of phenotypes) (Table 3).

**Table 3:** Number of phenotypical terms extracted per context and certainty

Context / Certainty	Negated	Not negated
Family history	179,938	522,009
Patient	5,007,517	12,988,474
Total number of terms	5,187,455	13,510,483

### Detailed expert evaluation

**Fig. 4:**
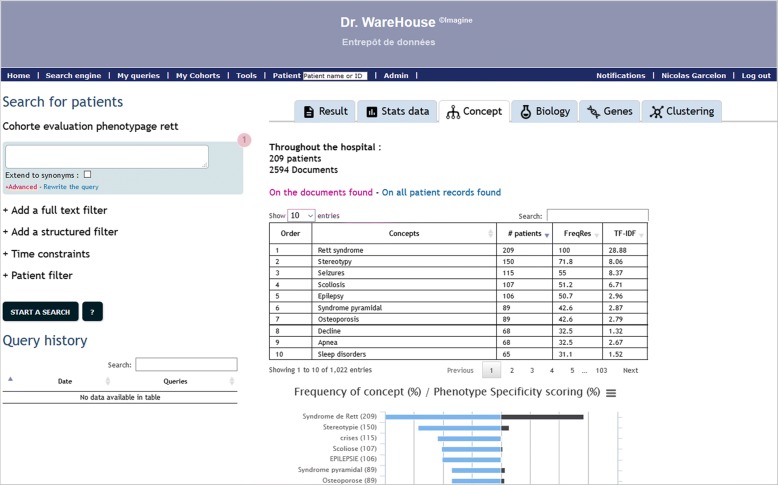
Screenshot of Dr. Warehouse and the concept tab for “Rett syndrome” query

The description of the data available in each cohort and the evaluation by the experts are detailed in Table 4. The Fig. 4 is a screenshot of the graphical user interface of Dr. Warehouse for Rett syndrome. The automated phenotyping identified an average of 768 phenotypical concepts associated to each disease. In contrast, the number of UMLS concepts found in Orphadata ranges from 16 for the Silver-Russell syndrome to 120 for the Lowe syndrome. APDS was not documented in Orphanet at the time of redaction of this article. Overall, the experts classified between 11 (SILVER-RUSSELL set, ranked by TF-IDF) and 41 (LOWE set, ranked by TF-IDF) of the top-50 results as relevant to the disease. The number of phenotypical concepts identified by the union of results obtained through ranking by frequency and ranking by TF-IDF ranges from 16 (SILVER-RUSSELL set) to 52 (DOCK8 deficiency and APDS sets).

**Table 4:** Description and evaluation of the 6 sets of patients

Sets	RETT	DOCK8 deficiency	LOWE	SILVER RUSSELL	BARDET BIEDL	APDS 1 and 2
Median age at visit (years)	8.2 [4.8–12.6]	11.4 [9.3–14.1]	12.8 [5.8–20.3]	2.4 [0.8–5.4]	15.7 [10.1–41.5]	12.8 [7.7–18.6]
Median follow up (years)	2.6 [0–4.9]	3.1 [0.3–9]	6.6 [3–10.3]	2 [0.8–4.7]	2 [0.1–6.6]	7.5 [4.8–8.6]
# Patients	209	15	23	50	53	23
# Documents	5034	3296	1325	1133	1317	2337
Phenotypes extracted, not negated and in patient context						
# Phenotypes	18,538	6886	5281	6563	6345	9716
# distinct Phenotypes	1022	706	577	738	801	710
Evaluation by experts in the Top50 phenotypes						
Medical Experts	NBB	CP	RS	JA	RS	NM
# Phenotypes ranked by Freq	31	36	36	16	17	39
# Phenotypes ranked by TF-IDF	38	37	41	11	12	37
# Phenotypes Freq union TF-IDF	42	52	50	16	19	52
# Phenotypes Freq intersect TF-IDF	28	22	28	11	11	25
Average Precision, ranked by Freq	0.86	0.91	0.88	0.55	0.66	0.83
Average Precision, ranked by TF-IDF	0.91	0.84	0.90	0.49	0.52	0.83

The Mean Average Precision was 0.79 for results ranked by Frequency and 0.75 for results ranked by TF-IDF. An additional file shows in detail the Top50 phenotypical concepts extracted for each cohort [see Additional file 1].

### Comparison with Orphadata

The comparison with Orphadata is detailed in Table 5. The limitation to French terms resulted in a reduction of an average of 16 phenotypes, corresponding to an average of 39% of the UMLS concepts (max: 63%, min 21%).

**Table 5:** Comparison with Orphadata

	RETT	DOCK8	LOWE	SILVER RUSSELL	BARDET BIEDL	APDS
# Concepts HPO Orphadata (English)	39	18	120	16	25	–
# Concepts HPO Orphadata (French) [A]	31	10	76	6	17	–
# UMLS distinct phenotypes extracted [B]	1022	706	577	738	801	710
# [A] intersection [B] (coverage)	22	7	50	6	14	–
% [A] intersection [B] / [A] (coverage %)	0.71	0.70	0.66	1.00	0.82	–

We obtained the best coverage for the SILVER RUSSEL set with 100% of the Orphadata phenotypes present in the phenotypes of the patient set. The lowest coverage was found for the LOWE set with 66% of the 76 Orphadata phenotypes present in the phenotypes of the patient set. The average coverage for all the patient sets was 78%.

In the six diseases studied, 2.8% of Orphadata concepts do not belong to the semantic types used for the automated phenotyping. For example, “Dislocated hips” (HP:0002827) is part of the description of Lowe syndrome in Orphadata and is assigned to the semantic type “Injury or Poisoning” in the UMLS.

Among the phenotypical concepts in Orphadata, 41 are not represented in the patient sets phenotypical concepts.

## Discussion

### Findings and practical significance

Our method of automated extraction of phenotypes from narrative reports in a clinical data warehouse can be useful even in the context of rare disease with a low number of patients. Indeed, 4 of the 6 examples showed results above 83% in terms of Average Precision in the top-50 phenotypes based on the experts’ evaluation. It was above 49% for the 2 remaining examples. It means that the extracted phenotypes are meaningful and can be used as baseline in diverse situations: enrichment of the phenotypic description of diseases, rapid exploration of the phenotypes in a population, or assisting the experts in the identification of phenotypes of interest.

Our approach can be used to enrich existing phenotypic description of rare diseases. For example, osteoporosis was significantly associated with Rett syndrome in the Necker data warehouse. The association is present neither in Orphanet nor in OMIM. It is however described in six articles in Medline [24–29].

Moreover, this method enables a quick exploration of phenotypes in a population. This feature is especially meaningful in the context of rare diseases for which the information may be scarce. In a research context, we have shown with the six examples, that our method was able to automatically display the phenotypes associated with rare diseases in a cohort of patients. The same approach could be used to look for undescribed phenotypes associated with new mutations (using gene names as a query for example or a series of patients selected manually). The Necker hospital and *Imagine* Institute collaborate actively to increase the knowledge on rare diseases and the phenotype explorer from the CDW is used on a daily basis by the staff to support translational research: When a geneticist discovers a new mutation, the exploration of the documents gathered from patients presenting the mutation in the CDW can support the description of the associated phenotypes. For example, the phenotypes associated to APDS 1 and 2 could provide basis for the description of the syndrome.

DrWH may also be used to assist experts in the identification of phenotypes of interest. After a careful review and comparison with other cohorts, such associations could be used to enrich online reference resources. Moreover, the method is easily reproducible, and the comparison of phenotypes coming from a variety of clinical data warehouses can provide candidates (union of the candidate phenotypes) or reinforce the interest on specific candidate phenotypes (using the intersection of different submissions).

In addition, the prevalence of signs and symptoms for a given disorder can be estimated using the frequencies provided by DrWH. Our method can provide the clinicians with an estimated prevalence of phenotypes in addition to the associations. In our running example Rett syndrome, “stereotypy” had a prevalence of 71.8%, consistent with Orphanet (Very frequent 80–99%); similarly “scoliosis” had a prevalence of 51.2%, vs. frequent (30–79%) in Orphanet. Conversely, the prevalence of “apraxia” in DrWH was 12.9%, whereas apraxia is considered very frequent (80–99%) in the Rett syndrome by Orphanet. A more precise estimation of the frequency would require considering not only single phenotypical concepts but also group of semantically close phenotypes.

### Limitations

#### Comparison to a gold standard and, interoperability issues

It was complex to perform an automated evaluation of phenotypes found by DrWH by comparison to a gold standard (e.g. Orphanet with Orphadata).

(1) The extraction of the phenotypical concepts was based on the French terms from the UMLS. However, the coverage of French term is limited compared to the extent of the English counterpart, knowing in particular that the French version of HPO was not available in the UMLS 2017AA. For example, in Orphadata the Rett syndrome is associated with 39 phenotypical concepts, of which only 31 exist in French in the UMLS (Table 5). The difference is more dramatic with the Lowe syndrome: for 120 phenotypical concepts, only 76 have a French counterpart. Our automated exploration is based on the use of medical terminologies in French, and DrWH cannot recognize a phenotypical concept that is not present in French. For example, Triangular Face (HPO: HP:0000325, UMLS: C1835884) is a sign associated to Silver-Russell syndrome in Orphadata and is absent from the UMLS concepts extracted from the corresponding set. Nevertheless, 27 patients of the SILVER-RUSSELL syndrome set have the string “face triangulaire” in their narrative records according to a full text search, but the concept “Triangular Face” does not exist in French in the UMLS. Despite this limitation, the current version of DrWH enables nonetheless relevant explorations, and allows the discovery of phenotypes of interest. The limited coverage of French terms compared to English limits our ability to identify concepts in free-text, but is also a limitation in our evaluation (which tends to underestimate the performance of the method). The integration of new terminologies (with a French translation) provided with mapping to UMLS, or integrated in the UMLS will reduce the gap between English and non-English terms. The recent increase of interest for non-English Natural Language Processing is a step forward in that direction.

(2) The granularity between phenotypical concepts extracted from Orphadata and DrWH may differ (e.g. a very precise term can be identified in DrWH whereas a more general term is present in Orphadata). This issue cannot be addressed by a simple hierarchical reasoning given that phenotypical concepts may be related semantically, but not identical nor hierarchically linked (e.g. *Hypotonia (C0026827)* vs *muscle weakness (C0151786)*).

(3) We solely used an exact match strategy (with text normalization) to recognize phenotypical concepts in the reports. Our method does not handle terms presenting the words in a different order (e.g. renal acute injury versus acute renal injury would not match). The presence of multiple synonyms in source terminologies might limit the impact of this strategy to a certain extent. However we intend to upgrade our phenotype recognition strategy to allow more flexibility in the recognition of phenotypical concepts.

(4) Some phenotypical concepts are not in the semantic types that we used for the automated phenotyping. In Orphadata, “autoagression” is a sign associated to Rett syndrome. We found 10 patients in the RETT set with “automutilation” in their narrative reports, but this concept is in the semantic type “Injury or Poisoning”.

#### Study population

Our warehouse hosts data produced by a children hospital, and therefore, phenotypes can be different from adult patients (for example, Alzheimer disease is not represented in pediatrics). However, patients with rare diseases may be followed-up in our institution even during adulthood, enabling an extended longitudinal data collection. Longitudinal follow-up makes it possible to observe the age of apparition of the phenotypes and reconstruct the natural history of rare diseases.

### Related work

#### Information extraction

Several approaches have been developed to recognize UMLS concepts, or terminology terms from free-text records. Savova et al. [30] developed cTAKES, an open source modular system of pipelined components combining rule-based and machine learning techniques. cTAKES aims at the extraction of information from the clinical narratives. Despite development in other languages [31, 32], most of the open source clinical Natural Language Processing systems have been developed for the English language (MedLee [33], MetaMap [34], HITex [35]). Many challenges have helped to test and assess the different tools and methodologies. In non-English languages, less out-of-the-box tools and less learning datasets are available to work with text. More recently a challenge was dedicated to the extraction of information in multiple language medical documents (including French) [36].

### Narrative reports versus coded data

We have shown that text exploration of clinical reports can provide phenotypes of interest. Whereas structured databases are particularly adapted for the collection of data regarding well documented diseases, clinical report based exploration enables the secondary use of data collected during care. Such approaches allow the development of learning health systems in which there is a bidirectional relation between routine care data and research. In addition, patient generated data could be integrated and mined along with the EHRs [37].

We plan to conduct additional studies by comparing our results with the French national rare diseases registry [38].

### Scalability

The Phenotype Explorer of Dr. Warehouse enables the exploration of millions of clinical narratives in a simple manner. The algorithm is optimized to display the phenotype analysis of thousands of documents quickly, and limited expertise is needed to write and execute queries. The queries demonstrated in this study only took a few seconds to run, enabling a real time exploration of the data. The expert user can easily sort the associated phenotypes according to their need, depending on the use case.

## Conclusion

Clinical Data Warehouses can be used to perform Next Generation Phenotyping, especially in the context of rare diseases. We have developed a method to detect phenotypes associated with a group of patients using medical concepts extracted from free-text clinical narratives. There are still hurdles to overcome with terminologies in non-English languages, however experts’ evaluation suggests that the phenotypes identified using the Frequency and TF-IDF scores can be useful to populate knowledge bases in addition to literature mining.

## Additional file

Additional file 1: Extracted phenotypical concepts per cohort. For each cohort, we list the top50 concepts ranked by Frequency and TF-IDF. The first column is the UMLS code of the phenotypical concepts, the second column is the French preferred terms, the third column is the English preferred terms, the fourth column is the frequencies score (FREQ), the fifth column is the TF-IDF score, the sixth column is the rank of the concept sorted by the frequency score, the seventh column is the rank of the concept sorted by the TF-IDF score and the eighth column is the expert evaluation (1: relevant concept, 0: none relevant concept). (XLS 93 kb)
